# Tailoring plasmonic size in Au/WO_3_ photonic crystals for photoelectrochemical water splitting and pharmaceutical degradation

**DOI:** 10.1039/d5ra04834f

**Published:** 2025-11-04

**Authors:** Maria-Athina Apostolaki, Marios-Konstantinos Christoforou, Elias Sakellis, Polychronis Tsipas, Vassilis Psycharis, Spiros Gardelis, Vlassis Likodimos

**Affiliations:** a Section of Condensed Matter Physics, Department of Physics, National and Kapodistrian University of Athens, University Campus 15784 Athens Greece vlikodimos@phys.uoa.gr; b Institute of Nanoscience and Nanotechnology, National Center for Scientific Research “Demokritos”, Agia Paraskevi 15341 Athens Greece

## Abstract

Integrating plasmonic nanoparticles (NPs) into semiconductor metal oxides and structuring them as photonic crystals have been two effective strategies to develop robust photo(electro)catalysts with improved light harvesting and suppressed electron–hole recombination. In this work, Au-decorated WO_3_ inverse opal photoanodes were engineered to synergistically exploit plasmonic, photonic, and charge transfer effects for enhanced photoelectrochemical water splitting and the degradation of pharmaceutical pollutants. The WO_3_ inverse opal scaffolds, fabricated *via* colloidal co-assembly, functioned as visible light photonic crystals, enabling slow photon effects by aligning their photonic band gap with the absorption edge of WO_3_ and the localized surface plasmon resonance (LSPR) of Au NPs. Au NPs of varying sizes (5–80 nm) were incorporated post-synthetically to tailor plasmonic behavior and band alignment at the Au–WO_3_ metal–semiconductor heterojunction. Photoelectrochemical measurements revealed optimal photocurrent generation for 20 nm Au NPs, driven by near-field enhancement and improved carrier generation, while 5 nm Au NPs exhibited the highest photoelectrocatalytic activity in ibuprofen degradation, facilitated by a favorable Fermi level shift and efficient interfacial electron transfer. This work highlights the importance of size-engineered plasmonic particles integrated into photonic crystal frameworks for the rational design of multifunctional photoelectrodes in solar energy conversion and environmental remediation.

## Abstract 2

Combination of plasmonic size-dependent near-field enhancement and electron transfer with slow photon effects in Au/WO_3_ inverse opal photoanodes boosts PEC and photoelectrocatalytic performance.

## Graphical abstract

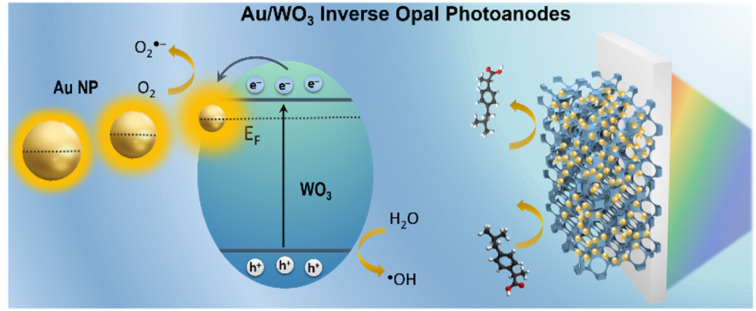

## Introduction

Semiconductor photocatalysis and photoelectrochemical (PEC) processes have gained increasing attention as environmentally friendly and energy-efficient technologies to address both the global energy crisis and environmental remediation challenges.^1–3^ Progress in this field depends critically on the development of robust photo(electro)catalysts, enabling efficient absorption of solar light to generate electron–hole pairs and drive redox reactions such as water splitting and the decomposition of organic pollutants with minimal secondary contamination. Metal-oxide semiconductors have been widely investigated as promising, environmentally benign materials for solar-to-chemical energy conversion applications due to their stability and abundance,^4^ whose PEC performance is, however, compromised by weak light harvesting as well as poor charge carrier separation and transport.^5^

To overcome such limitations, a powerful strategy is to integrate plasmonic metal nanoparticles (NPs) with the semiconductor photocatalysts.^6^ Noble-metal NPs (especially Au and Ag) exhibit strong localized surface plasmon resonances (LSPR) in the visible range, which depend on the NP size and shape as well as the dielectric environment and the degree of NP aggregation,^7–9^ creating intense local electromagnetic fields and generating energetic (“hot”) carriers upon illumination.^10^ These plasmonic NPs can enhance the photocatalytic activity by several complementary mechanisms based on the radiative and non-radiative LSPR decay: (i) near-field electromagnetic-field enhancement (boosting light absorption in the semiconductor), (ii) increased light scattering and optical path length, (iii) injection of hot electrons from the metal into the semiconductor (hot electron injection, HEI), and (iv) nonradiative dipole–dipole energy transfer to the semiconductor (plasmon-induced resonance energy transfer, PIRET).^6,11,12^ Gold is the material of choice for many plasmonic photocatalysts because of its chemical inertness, strong and tunable LSPR, and proven ability to increase visible-light absorption and charge separation in a wide range of semiconductors.^13^ Accordingly, Au NPs have been integrated with benchmark TiO_2_,^14–17^ ZnO,^18,19^ Fe_2_O_3_,^20,21^ and BiVO_4_ (ref. 22) metal oxide photoelectrodes, yielding notable advances in PEC H_2_ generation and photocatalytic wastewater treatment.

Among n-type metal oxide photoanodes, tungsten trioxide (WO_3_) combines a suitable energy bandgap (2.6–2.8 eV) with excellent carrier mobility (10–40 cm^2^ V^−1^ s^−1^) and long hole-diffusion length (150–500 nm).^23,24^ It is chemically stable in acidic electrolytes, strongly oxidizing, non-toxic, and inexpensive.^25^ Despite these advantages, pristine WO_3_ suffers from rapid electron–hole recombination, limited absorption beyond *ca.* 460 nm, and a relatively low conduction band potential for reductive processes (0.3–0.7 V *vs.* NHE), which restrict its PEC efficiency. Decorating WO_3_ with Au NPs may extend its optical absorption into the visible range and facilitate interfacial charge separation, improving photocatalytic pollutant degradation,^26,27^ as well as oxygen and hydrogen evolution.^28,29^ Reported enhancements have been related to PIRET and the co-catalyst action of Au NPs,^29^ near-field amplification^30^ and HEI^31^ for small Au NPs indicating that the enhancement pathway is determined by the interplay of the size-dependent Au plasmonic response and the metal–semiconductor band alignment.

Beyond material composition, the photocatalyst's architecture plays a critical role in determining its light-harvesting efficiency and charge transport dynamics.^32,33^ Three-dimensional (3D) photonic crystals, particularly bottom-up fabricated inverse opal (IO) structures, present a highly ordered macroporous network that simultaneously facilitates photon capture and mass transport.^34^ The periodic arrangement of voids in IO structures provides a structurally favourable scaffold for the incorporation of plasmonic NPs, enabling the formation of localized “hot spots” that greatly intensify the electromagnetic field at the metal–semiconductor interface.^35–37^ Moreover, the photonic bandgap (PBG) of IO structures induces slow-light effects at its band edges, effectively prolonging photon residence time within the material,^38,39^ and improving light collection in spectral regions where the semiconductor's absorption is weak. In addition, the large internal surface area and open porosity of the IO network promote reactants diffusion and facilitate electrolyte penetration, further promoting to photocatalytic activity. For wide-band-gap TiO_2_ IO photocatalysts, the synergy between slow photon modes and Au LSPR has resulted in photocatalytic gains.^40–43^ Similar improvements have been reported in visible-light-responsive BiVO_4_ IO photocatalysts through plasmonic decoration, where PBG engineering enabled the synergy of slow photon-enhanced LSPR excitation and HEI.^44–46^ However, recent studies highlight the key influence of charge transfer processes at the metal–semiconductor heterojunction, dictated by the underlying band alignment, on the photocatalytic performance.^35,47^

In this work, the interplay between plasmonic enhancement, charge transfer, and photonic amplification was investigated in nanostructured Au/WO_3_ IO photoanodes, fabricated *via* a colloidal co-assembly approach, in order to optimize light harvesting and charge separation for PEC and photoelectrocatalytic applications. A suitable WO_3_ photonic crystal film was accordingly selected in order to enable spectral overlap between the low and high PBG edges of the IO structure with the absorption edge of WO_3_ and the LSPR of Au NPs, respectively. Post-synthetic decoration of the nanocrystalline WO_3_ IO scaffold was carried out by using Au NPs with diameters ranging from 5 to 80 nm, which allowed to explore size-dependent plasmonic effects and modulate the band alignment at the Au-WO_3_ nanoscale heterojunction. The composite films were evaluated on both photocurrent generation and photoelectrocatalytic degradation of ibuprofen (IBU), a highly recalcitrant nonsteroidal anti-inflammatory drug of emerging concern in water treatment.^48^ Markedly enhanced performance was invariably observed for the smaller Au NPs reflecting the near-field enhancement and improved electron–hole pair generation, with a minor HEI contribution. In addition, PEC measurements and radical scavenger experiments in IBU degradation pointed to a significant role of size-dependent Fermi level shifts in Au NPs, facilitating electron transfer at the Au-WO_3_ heterojunction. The structure–property relationships obtained in this study offer insights for the rational design of advanced plasmonic–photonic photoanodes aimed at efficient solar energy conversion and environmental remediation.

## Experimental

### Au/WO_3_ inverse opal photoelectrodes fabrication

**Scheme 1:**
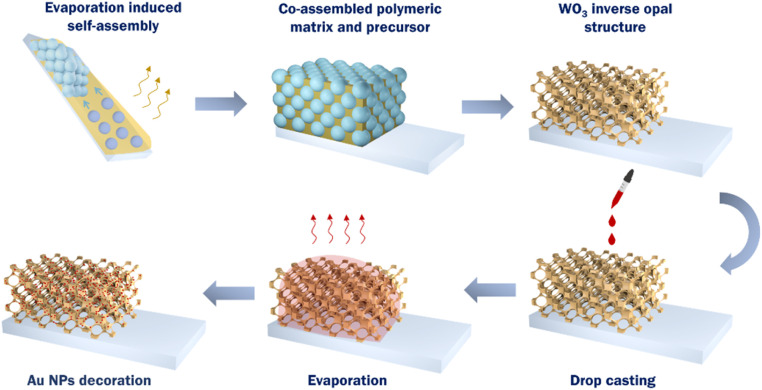
Schematic of the fabrication process of Au/WO_3_ IO photoelectrodes.

WO_3_ IO films were deposited on conductive glass substrates using the evaporative co-assembly of the polymer templating spheres with the metal oxide precursor (Scheme 1). Particularly, FTO (fluorine-doped tin oxide – FTO, thickness 2.2 mm, surface resistivity 7 Ω sq^−1^, Sigma-Aldrich) glass substrates were cleaned with Hellmanex™ III (Sigma-Aldrich) and ultrasound sonicated in acetone (≥99.5%, Honeywell Riedel-de Haën) and ethanol (EtOH, absolute ≥99.8%, Honeywell Riedel-de Haën). The FTO substrates were suspended almost vertically into beakers containing 8 mL of 0.125 wt% monodisperse poly(methyl methacrylate)-PMMA sphere suspension with mean diameter of 261 nm (colloidal dispersion of 5% solids (w v^−1^) in deionized water, 6 nm standard deviation, 2.4% CV, Microparticles GmbH) and 0.05 mL of WO_3_ precursor, prepared by dissolving 0.2 g ammonium metatungstate hydrate salt (AMT, Alfa Aesar) (NH_4_)_6_W_12_O_39_·*x*H_2_O in 1 mL H_2_O, 0.12 mL of 0.1 M citric acid (≥99.5%, Alfa Aesar) and 0.6 mL EtOH.^49^ The solvent evaporated at 55 °C and subsequently the dry films, comprising the infiltrated precursor within the opal interstices, were calcined at 450 °C for 2 h in air at heating rate 1 °C min^−1^ to remove the PMMA matrix and crystallize WO_3_ in the IO structure (Scheme 1). Au NPs of 5, 20, 50 and 80 nm diameter (stabilized suspensions in citrate buffer, optical density 1, Sigma-Aldrich) were deposited by drop casting on the films surface and dried at 60 °C for 30 min, yielding the Au/WO_3_ IO photocatalysts.

### Film characterization

The Au/WO_3_ films morphology and phase composition were investigated using a FEI Quanta Inspect scanning electron microscope (SEM) along with an energy-dispersive X-ray analyzer (EDX) and a FEI Talos F200i scanning transmission electron microscope (TEM) operating at 200 keV, equipped with a windowless energy-dispersive spectroscopy microanalyzer (6 T/100 Bruker). Powder X-ray diffraction (XRD) measurements were collected on a SmartLab Rigaku *θ*/*θ* Bragg–Brentano diffractometer (Cu Kα, 0.03° step, 11 s per step). Raman spectroscopy was performed using a LabRAM Soleil Horiba Raman microscope with 532 nm excitation, focused by a 100× (NA = 0.9) objective on the films' surface. X-ray photoelectron spectroscopy (XPS) was conducted using a PHOIBOS 100 (SPECS) analyzer with an Mg Kα (1253.6 eV) source. The spectrometer was calibrated using clean Ag, Cu, and Au, and binding energies were referenced to the C 1s peak at 284.8 eV. Spectra were fitted using XPS Peak Fit with Shirley background subtraction. Ultraviolet photoelectron spectroscopy (UPS) was performed with He I (21.22 eV) excitation. Optical properties were studied by specular and diffuse reflectance measurements on a Cary60 UV-vis spectrometer using a 15° specular and a fiber-optic diffuse reflectance accessory, respectively. A UV-enhanced Al mirror and a Halon reference were used for background determination. PEC measurements were carried out using a CS350 potentiostat/galvanostat (Corrtest Instruments) in a three-electrode system consisting of the IO films on FTO substrates as working electrodes, with a Pt foil as counter and Ag/AgCl as reference electrodes, immersed in 0.1 M NaHCO_3_ aqueous electrolyte and irradiated by UV-vis light provided by a 300 W Xe lamp, delivering 100 mW cm^−2^ measured by a thermal sensor power meter (PM160T, Thorlabs). Linear sweep voltammetry was performed at a potential scan rate of 10 mV s^−1^ and the photoconversion efficiencies (*η*) were calculated as a function of the applied potential by *η* = *J*[mA cm^−2^] × (1.23 – *V*_RHE_)/*P*[mW cm^−2^], where *J* is the photocurrent density at applied potential *V*_RHE_ and *P* is the total power density of incident light. Incident photon-to-current efficiency (IPCE) was measured at 1.23 V *vs.* RHE potential using a 1 kW Xe lamp, an Oriel 77 200 1/4 monochromator and a calibrated Si photodiode and IPCE was calculated according to the formula: IPCE (%) = (1239.8 [*V* nm] × *J*[mA cm^−2^])/(*λ*[nm] × *P*[mW cm^−2^]) × 100%, where *J* is the photocurrent density, *λ* is the wavelength of incident light and *P* is the monochromatic light power density. Electrochemical impedance spectroscopy (EIS) was performed in the frequency range of 10^5^–10^−2^ Hz with ac amplitude of 10 mV for Nyquist plots. Mott–Schottky measurements were conducted at 500 Hz with a scan rate of 10 mV s^−1^. Flat band potentials *V*_fb_ were determined from Mott–Schottky plots (1/*C*^2^*vs.* applied potential) using the equation:1
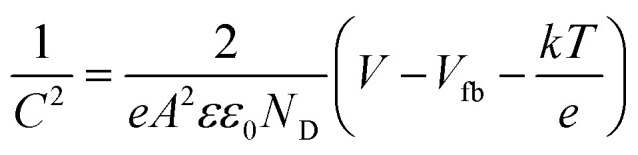
where *C* is the space-charge capacitance, *e* is the elementary charge, *A* is the electrode area, *ε*∼2000 is the WO_3_ relative permittivity at 500 Hz,^50^*ε*_0_ is the vacuum permittivity, *T* is the temperature, and *k* is Boltzmann's constant. The applied potential *versus* Ag/AgCl reference electrode was converted to the reversible hydrogen electrode (RHE) scale using Nernst equation: *V*_RHE_ = *V*_Ag/AgCl_ + 0.059 pH + 0.205.

### Photoelectrocatalytic evaluation

Photoelectrocatalytic degradation experiments were also carried out in a three-electrode configuration under UV-Vis illumination (300 W Xe lamp, 100 mW cm^−2^) and applied bias of +1.5 V *vs.* Ag/AgCl. IO films of 3 cm^2^ area were vertically immersed in beakers containing 20 mL working solution with 0.1 M NaHCO_3_ as supporting electrolyte and 5 mg L^−1^ IBU (4-isobutyl-alpha-methylphenylacetic acid, 99%, Thermo Scientific Chemicals) as pharmaceutical model pollutant. The solution was stirred for 30 min under dark conditions with no external activation to achieve adsorption–desorption equilibrium. The solution pH was stabilized at 3 by dilute HCl (fuming, ≥37) favouring the electrostatic attraction between positively charged WO_3_ and negatively IBU molecules. Additional photoelectrocatalytic tests were carried out in the presence of 5 mM propanol-2 (IPA, HPLC, 99.8%), 5 mM formic acid and 1 mM 1,4 benzoquinone as hydroxyl radical (˙OH), hole (h^+^) and superoxide anion radical (O_2_˙^−^) scavengers, respectively.

## Results and discussion

### Structural properties

**Fig. 1:**
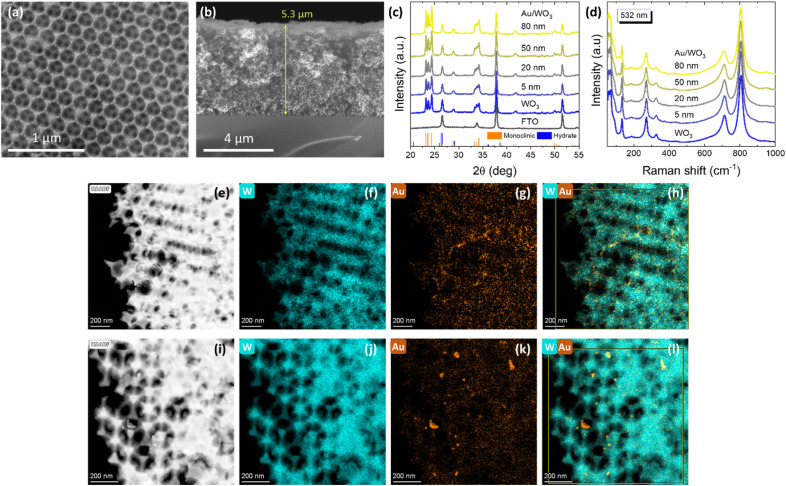
(a) Top-view and (b) cross-sectional SEM images of 20 nm Au/WO_3_ IO films, showing the periodic macroporous architecture. (c) XRD patterns and (d) Raman spectra at 532 nm excitation of the Au-decorated WO_3_ IO films compared to pristine WO_3_. The XRD patterns of the FTO substrate, *m*-WO_3_ (JCPDS 43-1035) and WO_3_·0.33H_2_O (JCPDS 35-0270) phases are also displayed in (c). (e–h) TEM image and corresponding EDX elemental maps (W, Au, and W/Au overlay) of WO_3_ IO films decorated with 5 nm Au NPs. (i–l) TEM image and EDX elemental maps for WO_3_ IO films decorated with 20 nm Au NPs.

The morphology of the Au/WO_3_ IO photonic crystal films was characterized by SEM, as shown in Fig. 1a and b. Top-view and cross-sectional SEM images reveal a well-ordered, periodic macroporous architecture with three-dimensionally interconnected walls, originating from the close-packed assembly of PMMA spheres and the simultaneous infiltration of the AMT precursor into the interstitial spaces. Upon calcination, decomposition of the PMMA opal template and crystallization of WO_3_ resulted in the periodic IO structure with high structural order. The resulting pore diameter was 195(5) nm, representing approximately 25% shrinkage from the original PMMA sphere diameter (261 nm), consistent with the polymer removal and densification of the metal oxide framework. The film thickness was ∼5.3(1) μm. Importantly, no discernible changes in the overall morphology were observed after decoration with Au NPs, indicating that the plasmonic modification preserved the structural integrity of the WO_3_ IO scaffold.

**Fig. 2:**
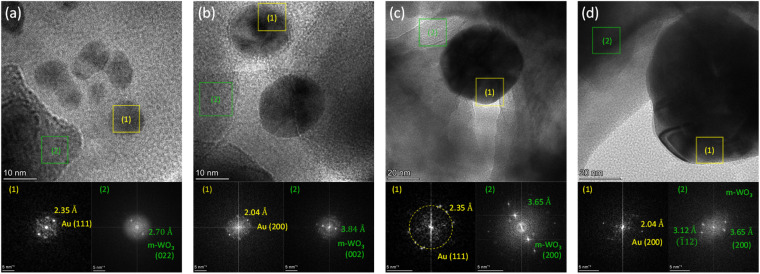
High-resolution TEM images of Au/WO_3_ IO films decorated with Au NPs of varying sizes: (a) 5, (b) 20, (c) 50, and (d) 80 nm. The lower panels show the corresponding FFT patterns, where Au NPs are identified by characteristic diffraction spots corresponding to the (111) and (200) Au fcc planes, embedded within the nanocrystalline *m*-WO_3_ matrix.

The crystalline phase of the Au/WO_3_ IO films was examined by XRD before and after Au NP decoration (Fig. 1c). All samples crystallized in the monoclinic WO_3_ (*m*-WO_3_) phase (JCPDS 43-1035, space group *P*2_1_/*n*), as evidenced by the characteristic low-symmetry splitting of the (002), (020), and (200) reflections at 23.2°, 23.7°, and 24.4°, respectively.^49^ A weak contribution from hydrated WO_3_·0.33H_2_O (JCPDS 35-0270) was barely detectable at ∼28.9°. Raman spectroscopy further confirmed the formation of *m*-WO_3_, irrespective of Au NP presence. All samples exhibited the characteristic vibrational modes of *m*-WO_3_, with prominent bands at 805 and 709 cm^−1^ corresponding to W–O–W stretching modes, and bands at 325 and 264 cm^−1^ attributed to W–O–W bending vibrations.^51^ In the low-frequency region, peaks at 58, 69, and 134 cm^−1^ were associated with lattice modes of the *m*-WO_3_ phase involving collective rotations of WO_3_ octahedra.^52^ A weak band observed near 950 cm^−1^ is assigned to the W=O stretching vibration, indicative of minor contributions from hydrated WO_3_.^53^ TEM combined with EDX analysis, confirmed the successful deposition of Au NPs on the WO_3_ IO framework (Fig. 1e–l). Elemental mapping showed the spatial distribution of W and Au, verifying uniform coverage of the WO_3_ skeleton by Au nanostructures. High-resolution TEM images (Fig. 2a–d) revealed Au NPs of varying diameters (5–80 nm) grafted to the WO_3_ framework. Fast Fourier Transform (FFT) analysis of the HRTEM images identified characteristic diffraction spots corresponding to interplanar spacings of 2.35 Å and 2.04 Å, consistent with the (111) and (200) planes of face-centred cubic (fcc) Au, respectively. Additionally, FFT patterns showed diffraction spots with interplanar spacings of around 2.7 Å and 3.7 Å, attributed to overlapping contributions from the (022), (2̄02), (202), (220) and (002), (020), (200) planes of *m*-WO_3_ (JCPDS No. 43-1035), respectively, confirming the preservation of the monoclinic phase after Au NP deposition.^54^

**Fig. 3:**
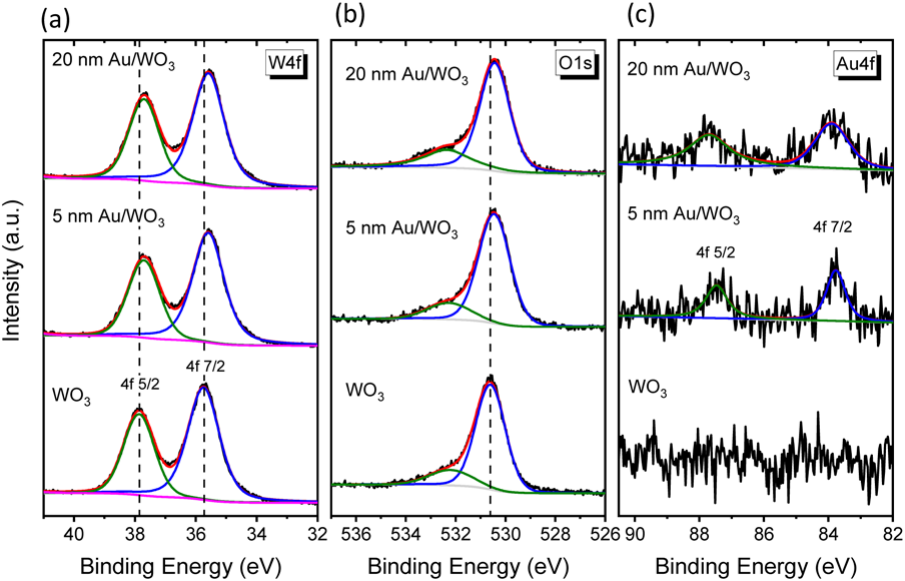
(a) W 4f, (b) O 1s, and (c) Au 4f core level XP spectra for the 5 and 20 nm Au/WO_3_ IO films compared to the pristine one.

The surface chemical composition and the successful deposition of Au NPs onto the WO_3_ IOs were examined by XPS (Fig. 3). Deconvolution of the W 4f spectrum for the pristine WO_3_ IO revealed two characteristic peaks at 35.7 and 37.8 eV, with a spin–orbit separation of 2.1 eV and an area ratio of 4 : 3, corresponding to the W 4f_7/2_–4f_5/2_ doublet of W^6+^ species.^31,49^ The O 1s spectrum exhibited a dominant peak at 530.6 eV, attributed to lattice oxygen, and a broader component at 532.2 eV associated with adsorbed oxygen species.^31^ The presence of Au NPs was confirmed by the Au 4f core-level spectrum, which showed peaks at 83.8 and 87.5 eV with a spin–orbit splitting of 3.7 eV, characteristic of the 4f_7/2_ and 4f_5/2_ levels of metallic Au^0^.^35^ Notably, both the W 4f and O 1s peaks in the Au/WO_3_ IO films shifted toward lower binding energies by approximately 0.15 eV compared with the pristine sample, indicating an upward shift of the Fermi level relative to the core levels and, consequently, a reduction in the work function (*vide infra*).

### Optical properties

**Fig. 4:**
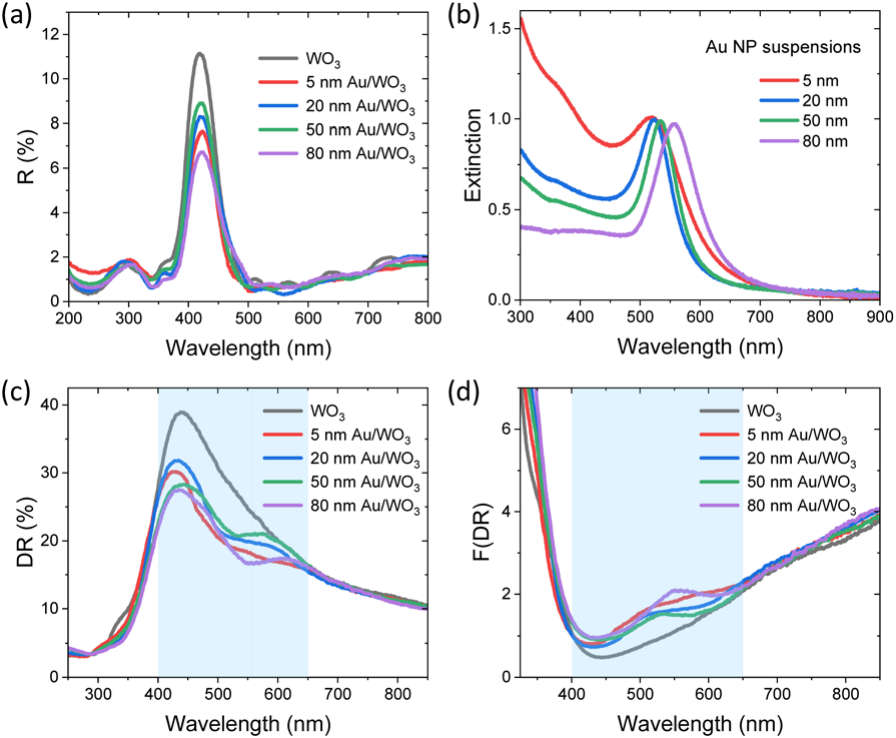
(a) Specular reflectance spectra of the WO_3_ IO films before and after Au NP decoration, showing the Bragg peak. (b) Extinction spectra of colloidal Au NPs with diameters of 5, 20, 50, and 80 nm, illustrating the size-dependent red-shift of the LSPR band. (c) Diffuse reflectance spectra, and (d) Kubelka–Munk transformed absorbance spectra F(DR) of pristine and Au/WO_3_ IO films. Shaded bands highlight changes of visible light absorption due to the LSPR-induced enhancement.

The optical properties of the Au/WO_3_ IO films were investigated to evaluate PBG formation and plasmonic effects. Specular reflectance (*R*%) spectra recorded at 15° incidence angle (Fig. 4a) confirmed the presence of a stop-band (incomplete PBG) along the [111] direction, evidenced by a strong Bragg reflection peak centred around 420 nm for all IO films. This characteristic peak arises from first-order Bragg diffraction by the periodic IO structure. Notably, post-deposition of Au NPs did not alter the spectral position of the stop-band, indicating that the IO architecture remained structurally intact. However, a decrease in reflectance intensity was observed, related to the LSPR absorption by the embedded Au NPs.

Using modified Bragg's law for first-order diffraction from the (111) fcc planes,^49^ the stop-band position in an aqueous medium was estimated at 491(14) nm. Assuming that the PBG width corresponds to the full-width at half-maximum of *ca.* 60 nm of the Bragg R peak (Fig. 4a),^55^ the stop band in water would roughly extend between 460–520 nm. The corresponding slow photon regions *i.e.* the spectral ranges where photon group velocity is reduced, which span approximately 20 nm at the PBG edges, were estimated. The red-edge slow photon region would lie between 520–540 nm, while the blue-edge region spans 440–460 nm. In this case, blue-edge slow photons overlap the intrinsic absorption edge of WO_3_ (*ca.* 450 nm, *vide infra*), enhancing electronic excitation, while red-edge slow photons spectrally match to the LSPR of Au NPs, amplifying plasmonic effects, and rationalizing the selection of the specific WO_3_ IO as support for the Au NPs. As shown in the extinction spectra of the colloidal Au NPs (Fig. 4b), the LSPR peak position red-shifts with increasing particle diameter, centred at approximately 519, 524, 535, and 556 nm for 5, 20, 50, and 80 nm Au NPs, respectively. This shift results from the breakdown of the dipole approximation in larger particles (>20 nm).^9^

Diffuse reflectance spectra of the pristine WO_3_ and Au-decorated WO_3_ IO films (Fig. 4c) reveal the fundamental absorption edge of the semiconductor at 450 nm, accompanied by a shoulder attributed to the photonic stop band. Upon Au NP deposition, the Au/WO_3_ films exhibited a size-dependent decrease in reflectance across the visible range (400–650 nm), following closely the LSPR extinction features of the colloidal Au NP suspensions (Fig. 4b). This light attenuation effect is related to plasmon-induced absorption and scattering, which enhances visible-light harvesting. The Kubelka–Munk transform spectra, F(DR), derived from the DR data (Fig. 4d) further confirm the LSPR-driven enhancement in optical absorption, showing a broad absorption band between 450 and 650 nm that mirrors the LSPR characteristics of the corresponding Au NPs. Notably, for the smallest Au NPs (5 nm), the absorption extended well beyond the expected LSPR region, suggesting the involvement of higher-order plasmonic modes. This red-shifted broadening is likely due to interparticle coupling effects arising from densely packed or aggregated Au NPs on the WO_3_ surface, which facilitate collective plasmonic resonances at longer wavelengths.^9,56^

### PEC evaluation

**Fig. 5:**
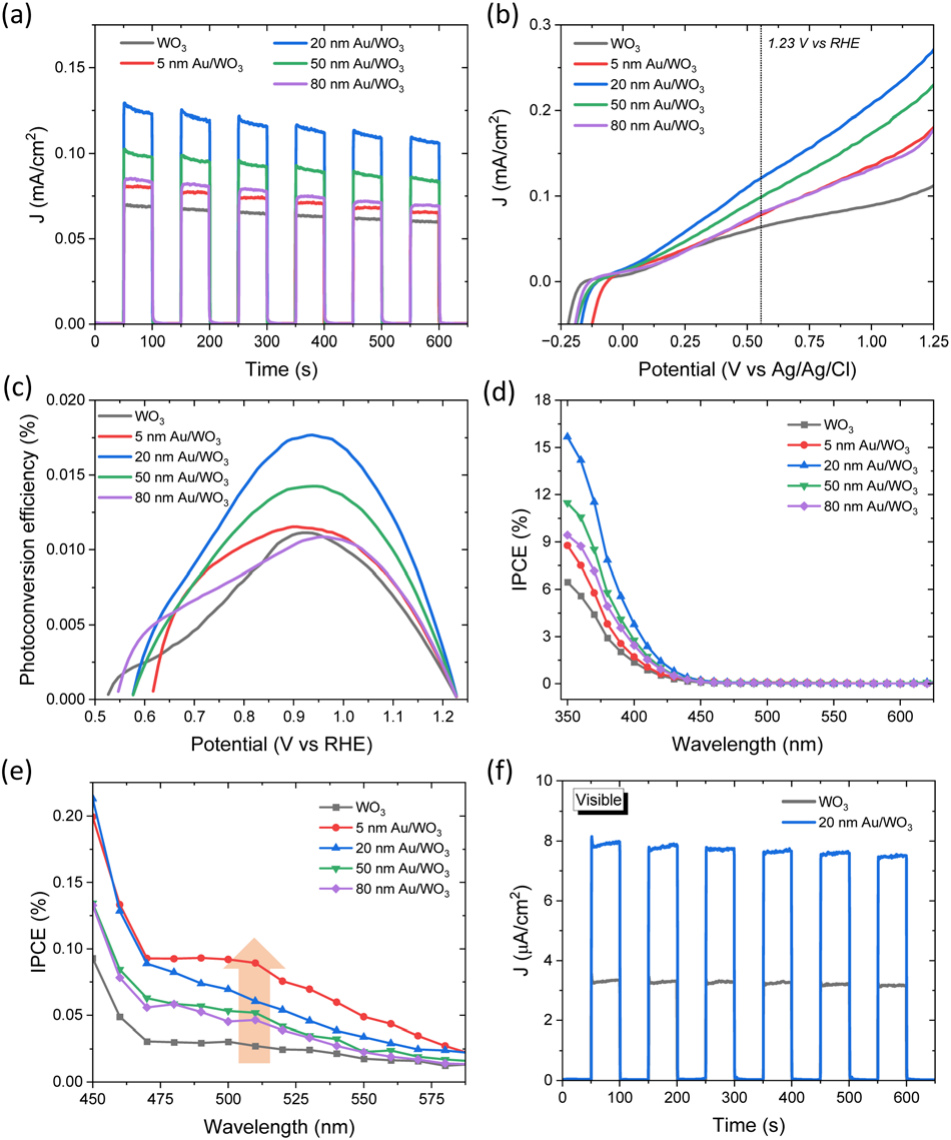
(a) Chronoamperometric *J vs. t* curves under chopped light, (b) linear sweep voltammetry, (c) photoconversion efficiencies, and (d) IPCE spectra for Au/WO_3_ IO photoanodes at 1.23 *V*_RHE_. (e) IPCE enhancement in the visible range for the Au/WO_3_ IO films. (f) Comparative photocurrent response for pristine WO_3_ and 20 nm Au/WO_3_ photoelectrodes under visible light (*λ* > 400 nm) illumination at 1.23 *V*_RHE_.

The PEC performance of Au-decorated WO_3_ IO photoelectrodes was assessed in 0.1 M NaHCO_3_ under chopped UV-Vis back-side illumination (50 s light–dark cycles), as shown in Fig. 5a. All Au/WO_3_ IO photoanodes exhibited enhanced photocurrent responses compared to pristine WO_3_ IO, with a clear dependence on the size of the Au NPs. The 20 nm Au/WO_3_ photoanode displayed the highest photocurrent density (*J*) of 0.12 mA cm^−2^ at 1.23 *V*_RHE_, followed by the 50 nm Au/WO_3_ (0.10 mA cm^−2^). In contrast, the 5 and 80 nm Au/WO_3_ IO electrodes exhibited only modest increases compared to 0.07 mA cm^−2^ of the unmodified WO_3_ IO. These trends were consistent with the linear sweep voltammetry profiles under illumination (Fig. 5b) and the corresponding photoconversion efficiencies (*η*) as a function of applied potential (Fig. 5c). Furthermore, control experiments (Fig. S1, SI) revealed a pronounced deterioration in photocurrent for both pristine WO_3_ and 20 nm Au/WO_3_ planar films, which were prepared by dip-coating the WO_3_ precursor onto FTO substrates in the absence of the colloidal template, followed by calcination under the same conditions. This observation clearly highlights the beneficial role of the IO photonic architecture in enhancing photoelectrochemical performance.

IPCE measurements were carried out at 1.23 *V*_RHE_ to explore the wavelength-dependent PEC response of the Au/WO_3_ IO photoanodes (Fig. 5d). The IPCE spectrum of pristine WO_3_ IO showed significant increase below ∼450 nm, in agreement with the WO_3_ absorption edge. Upon Au decoration, all photoelectrodes demonstrated enhanced IPCE within the same spectral window, with the highest gains observed for the 20 nm Au/WO_3_ IO, followed by the 50, 80, and 5 nm variants. Taking into account the progressive increase of the absorption cross section of metallic Au NPs with the decrease of particle size,^6,7^ these improvements can be primarily related to the local field enhancement mechanism, which promotes the generation of electron–hole pairs near the Au/WO_3_ interface. The increased scattering cross section with the increase of plasmonic NP size along with and the accompanying LSPR shift to longer wavelengths, complies well with the progressive moderation of IPCE enhancement for the 50 and 80 nm Au/WO_3_ IOs. However, while smaller Au NPs generally provide stronger absorption and field enhancement, the 5 nm Au NPs showed the weakest improvement, most likely due to the enhanced aggregation of the 5 nm NPs evidenced by the TEM analysis (Fig. 2a). The formation of Au NP clusters and the underlying interparticle interactions results in the broadening and red-shift the LSPR band beyond the WO_3_ absorption edge (Fig. 4d) leading to the moderation of near-field enhancement, which is a radiative process, and the ensuing PEC performance. Nevertheless, a slight rise in IPCE was observed in the 450–580 nm region (Fig. 5e), consistent with the Au NP LSPR band and suggesting a minor contribution from HEI into the WO_3_ conduction band. This contribution was most noticeable in the 5 nm Au/WO_3_ IO photoanode, in line with the higher intrinsic LSPR absorption efficiency of smaller Au NPs. Under visible-light-only illumination (*λ* > 400 nm), the 20 nm Au/WO_3_ photoanode showed a photocurrent more than double that of the pristine WO_3_ IO (Fig. 5f), further supporting the synergistic role of plasmonic enhancement. Overall, these results point to near-field electromagnetic enhancement as the dominant mechanism in improving PEC water splitting, particularly when the Au NPs are well-dispersed and spectrally matched with the semiconductor absorption and photonic stop-band edges.

**Fig. 6:**
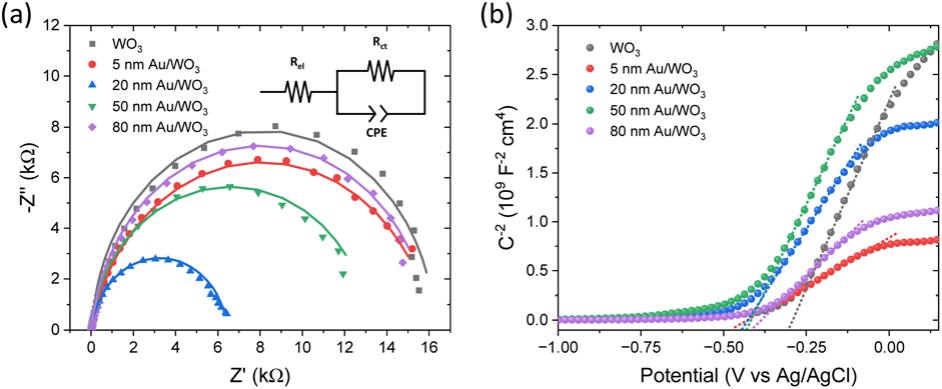
(a) EIS Nyquist and (b) Mott–Schottky plots for the Au/WO_3_ photoanodes. Solid lines in Nyquist plots show the best-fit curves to the modified Randles equivalent circuit.

EIS measurements were further performed to probe interfacial charge transfer dynamics. Nyquist plots (Fig. 6a) revealed semicircular profiles, whose curvature and radius varied with the Au NP size. These were modelled using a modified Randles equivalent circuit (inset, Fig. 6a) comprising a series resistance (*R*_el_), a charge transfer resistance (*R*_ct_) across the semiconductor–electrolyte interface, and a constant phase element (CPE), whose phase remains constant with frequency and the impedance is expressed as *Z*_CPE_ = [*Q*(*jω*)^*β*^]^−1^, where *Q* represents the capacitance of the oxide layer and the exponent *β* = 1 for ideal capacitance, appropriate for the porous morphology of the IO films.^57,58^ Among all samples, the 20 and 50 nm Au/WO_3_ electrodes exhibited the lowest *R*_ct_ values (Table 1), indicating significantly improved interfacial charge transport and reduced recombination, in line with their photocurrent performance. Conversely, the 5 and 80 nm Au/WO_3_ electrodes showed *R*_ct_ values comparable to that of pristine WO_3_.

**Table 1:** Parameters obtained by the analysis of Nyquist and Mott–Schottky plots for the Au/WO_3_ IO photoelectrodes

Film	*R*_el_ (Ω)	*R*_ct_ (Ω)	*Q* ( ×10^−4^)	*β*	*V*_fb_ (*V*_RHE_)
WO_3_	60.1	16 214.6	8.1	0.98	0.358
5 nm Au/WO_3_	8.8	16 193.0	7.0	0.88	0.222
20 nm Au/WO_3_	13.5	6471.9	10.8	0.92	0.225
50 nm Au/WO_3_	23.1	13 176.4	15.5	0.94	0.233
80 nm Au/WO_3_	34.6	16 021.3	10.4	0.94	0.271

Mott–Schottky analysis (Fig. 6b) confirmed n-type semiconducting behaviour for all samples. The flat-band potential *V*_fb_ for pristine WO_3_ was estimated at −0.29 V *vs.* Ag/AgCl. Notably, Au NP decoration induced a systematic negative shift in *V*_fb_, more pronounced for the smaller Au particles, suggesting a Fermi level shift towards more negative potentials, consistent with previous studies.^59,60^ These findings reinforce the role of Au NPs in enhancing charge separation and transfer at the metal–semiconductor interface, further contributing to the overall PEC performance.

### Photoelectrocatalytic performance

**Fig. 7:**
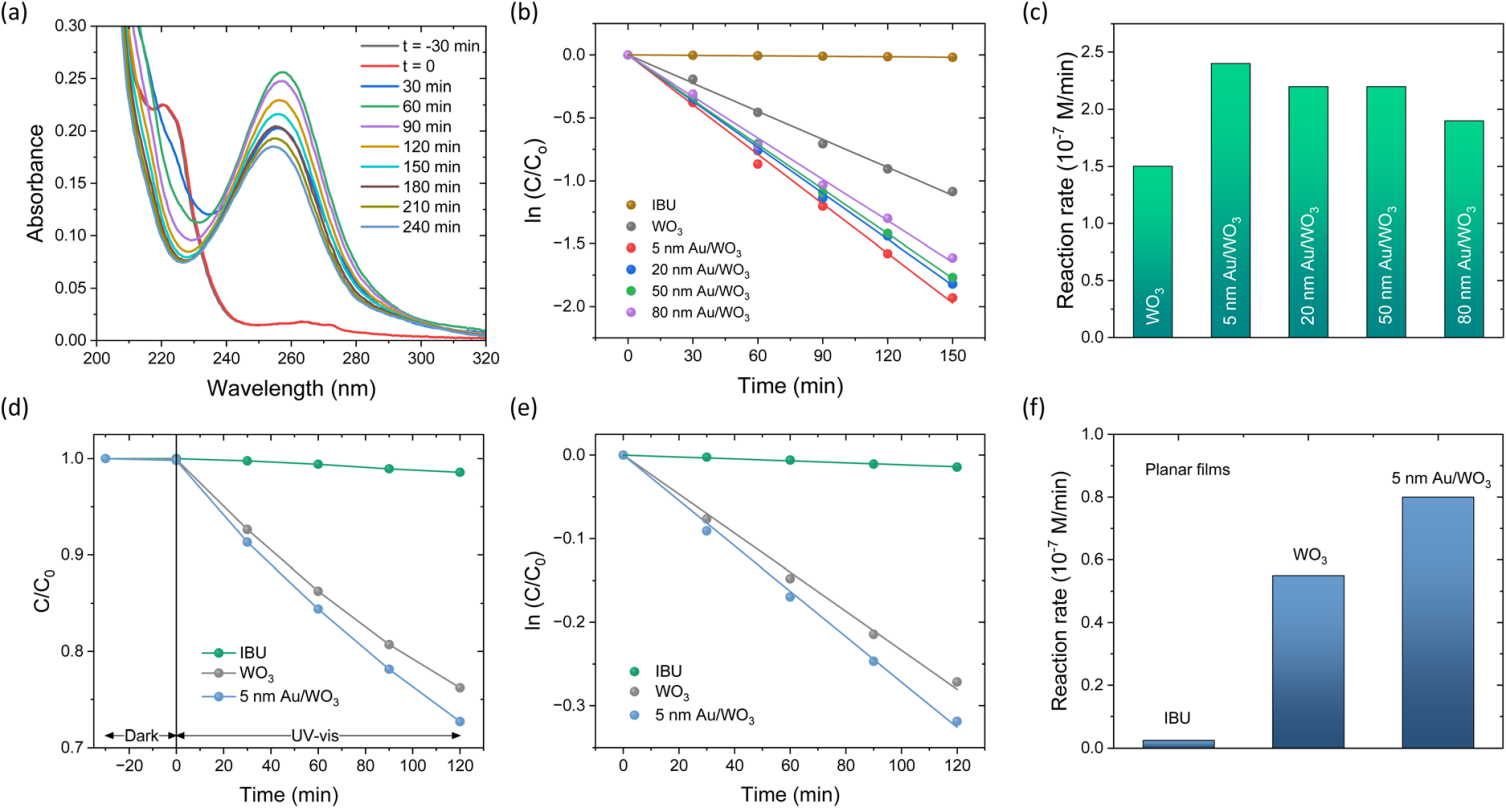
(a) Absorbance spectra evolution of IBU during degradation by 5 nm Au/WO_3_ IO in 0.1 M NaHCO_3_ supporting electrolyte at +1.5 V *vs.* Ag/AgCl under UV-Vis irradiation. (b) Pseudo-first-order kinetic plots and (c) corresponding reaction rates for the different Au/WO_3_ IO photoelectrodes. (d)–(f) Control IBU PEC degradation kinetics for planar WO_3_ and 5 nm Au/WO_3_ photoanodes under identical conditions.

The photoelectrocatalytic activity of the Au/WO_3_ IO films was assessed *via* the degradation of ibuprofen (IBU), a widely used anti-inflammatory pharmaceutical frequently detected as a contaminant in aquatic environments.^61^Fig. 7a illustrates the time-resolved UV-vis absorbance spectra of IBU during photoelectrocatalysis using the 5 nm Au/WO_3_ IO film in 0.1 M NaHCO_3_ electrolyte at +1.5 V *vs.* Ag/AgCl under UV-vis light. No evidence of appreciable IBU adsorption was observed on WO_3_ in the absence of light. Upon illumination, the characteristic aromatic absorption band of IBU at 222 nm progressively decreased, while a new band emerged at 256 nm within the first 30 min, corresponding to the formation of the intermediate by-product 4-isobutylacetophenone.^62,63^ The degradation kinetics were evaluated using plots of ln(*C*/*C*_0_) *versus* time (Fig. 7b), where *C*_0_ is the initial IBU concentration and *C* is the concentration at time *t*. The linear correlation indicates that the degradation followed pseudo-first-order kinetics, with the rate constant *k* derived from the slope. The reaction rates were calculated using *r* = *kC*_0_ to compare the catalytic performance of different photoelectrodes. Incorporation of Au NPs significantly enhanced the photocatalytic activity for all tested particle sizes. The highest degradation rate was observed for the 5 nm Au/WO_3_ IO film (Fig. 7c), followed by the 20 and 50 nm counterparts, while only a modest improvement was noted for the 80 nm Au/WO_3_ film. This size-dependent trend aligns with the variations in absorption and scattering cross-sections of Au NPs,^7^ which modulate light trapping and plasmonic enhancement effects.^6,12^ Moreover, control experiments on photoelectrocatalytic IBU degradation under identical conditions using planar WO_3_ and 5 nm Au/WO_3_ photoanodes (Fig. 7d–f) exhibited significantly lower reaction rates—approximately three times lower—than those of the corresponding IO-based electrodes. In addition, the relative enhancement in activity upon decoration with 5 nm Au NPs was smaller for the planar films, further corroborating the crucial role of the IO structure in promoting photocatalytic performance.

**Fig. 8:**
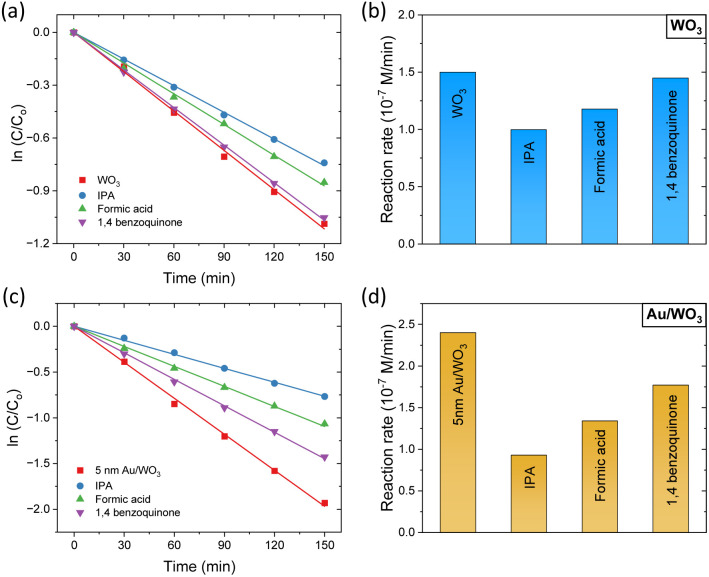
IBU photodegradation kinetics and reaction rates for (a) and (b) pristine WO_3_ and (c) and (d) 5 nm Au/WO_3_ IO photocatalysts in the presence of IPA, formic acid, and 1,4-benzoquinone radical scavengers in 0.1 M NaHCO_3_ supporting electrolyte at +1.5 V *vs.* Ag/AgCl under UV-Vis irradiation.

To gain mechanistic insight into the IBU degradation pathway, scavenger experiments were performed for the pristine WO_3_ and the 5 nm Au/WO_3_ IO photocatalysts using IPA, formic acid, and 1,4-benzoquinone as scavengers for hydroxyl radicals (˙OH), photogenerated holes (h^+^), and superoxide radicals (O_2_˙^−^), respectively (Fig. 8). The presence of IPA significantly suppressed the photocatalytic activity, indicating that ˙OH radicals are the dominant oxidative species in IBU degradation. Formic acid also caused a notable reduction in efficiency, highlighting the crucial role of valence band holes. This suppression was more pronounced for the Au-modified film, suggesting enhanced hole accumulation and stronger oxidative potential due to Au-induced charge separation and reduced recombination. In contrast, 1,4-benzoquinone had a negligible effect on the pristine WO_3_ catalyst, whereas appreciable reduction in activity was observed for the Au/WO_3_ photocatalyst. This implies that superoxide radicals (O_2_˙^−^) contribute to the reaction only in the presence of Au NPs, further supporting their role as co-catalysts that facilitate interfacial charge transfer and broaden the oxidative pathways in the photoelectrocatalytic process.

**Scheme 2:**
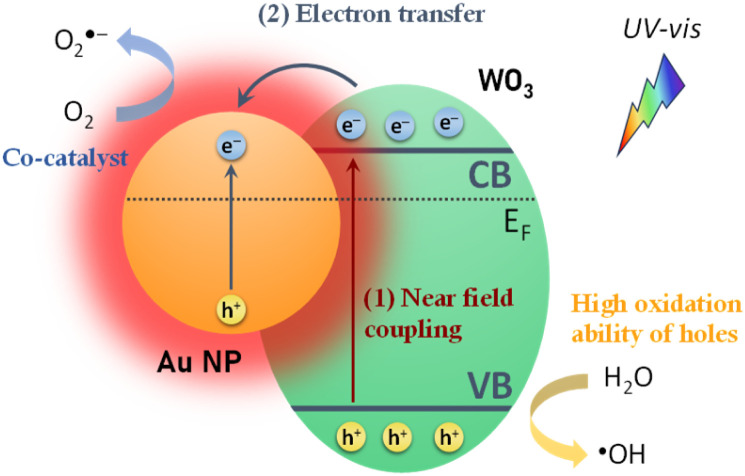
Schematic representation of the major enhancement mechanisms contributing to PEC and photoelectrocatalytic performance in the Au/WO_3_ IO system: (1) local electric field amplification near Au NPs, and (2) electron transfer from WO_3_ to Au, promoting charge separation and pollutant degradation.

Interestingly, the photoelectrocatalytic degradation activity of the Au/WO_3_ IO films exhibited a different size-dependence compared to their PEC water splitting performance (Fig. 5). While 20 nm Au NPs offered the highest PEC activity, the smallest 5 nm Au NPs led to the most efficient IBU degradation. This variation indicates the involvement of additional mechanisms beyond local field enhancement, which dominate under photoelectrocatalytic conditions and differentiate the behaviour of the system in pollutant degradation *versus* water oxidation. For PEC water splitting, the primary enhancement mechanism is attributed to the LSPR-induced local field amplification. When WO_3_ nanocrystals are in proximity to Au NPs, the intensified local fields significantly enhance light absorption and boost the generation of photoinduced electron–hole pairs (Scheme 2). The magnitude of this effect is proportional to the square of the local electric field intensity,^64^ resulting in a substantial increase in carrier generation, particularly within the spectral range corresponding to the LSPR bands, as evidenced by the IPCE spectra (Fig. 5d).

However, for the photoelectrocatalytic degradation of IBU, additional electronic effects play a key role. Mott–Schottky analysis (Table 1) revealed a systematic, size-dependent shift in *V*_fb_ potentials, suggesting that the Fermi level of Au NPs changes with particle size, thereby affecting the metal–semiconductor band alignment.^65,66^ The nature of this alignment determines whether a Schottky barrier forms at the metal–WO_3_ interface.^67^ For Au NPs with a work function (*ϕ*_M_) of 5.1 eV, potentially higher by ∼0.2 eV with citrate capping,^68^ a Schottky barrier with a height up to ∼0.7 eV could be expected when interfaced with the WO_3_ IO skeleton for which the electron affinity *χ*_S_ was reported to be 4.60 eV.^49^ However, literature reports indicate that decreasing the NP size reduces *ϕ*_M_, shifting upward the Fermi level of the Au NPs.^59,60^

**Fig. 9:**
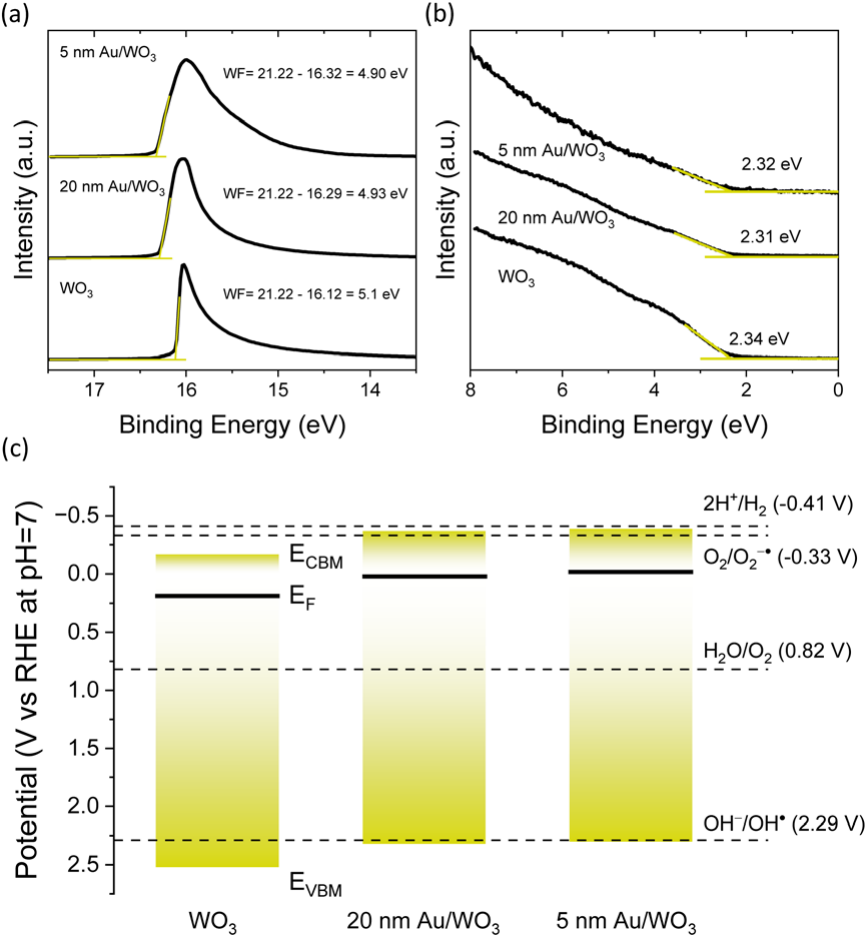
(a) Secondary electron cut-off and (b) valence band UP spectra for the 5 and 20 nm Au/WO_3_ IO films compared to the pristine one. (c) Energy band and redox potentials *vs.* RHE at pH = 7 derived for the IO films.

UPS was accordingly used to explore the electronic properties and band alignment at the Au–WO_3_ interface. The secondary electron cut-off (Fig. 9a) and valence band spectral regions (Fig. 9b) were used to determine the work function (WF) and valence band maximum (VBM) with respect to the Fermi level (*E*_F_) for the 5 and 20 nm Au/WO_3_ IO films compared to the pristine one. The obtained WF and *E*_F_–*E*_VBM_ together with the WO_3_ IO electronic band gap of 2.7 eV determined from indirect band gap Tauc plot (Fig. S2, SI), were used to construct the corresponding energy band diagram. A Fermi level shift towards the conduction band minimum (*E*_CBM_) was observed for the 5 and 20 nm Au/WO_3_ IO films, in agreement with the XPS analysis (Fig. 3) and negative *V*_fb_ shifts observed in the Mott–Schottky (Fig. 6b). This upward Fermi level shift, most prominent in the 5 nm Au NPs, aligns more closely, or even exceeds, the Fermi level of WO_3_ nanocrystals (Fig. 9c). As a result, band bending is reduced and the Schottky barrier suppressed, promoting direct electron transfer from the WO_3_ conduction band to the Au NPs. This charge transfer facilitates electron scavenging by the metallic NPs, reinforcing their co-catalyst role and enhancing charge separation at the interface. Furthermore, this band alignment shifts the CBM closer to the O_2_/O_2_^−^ reduction potential (−0.33 V *vs.* NHE),^69^ enabling the generation of superoxide radicals (O_2_˙^−^) (Fig. 9c), especially for the 5 nm Au/WO_3_ IOs presenting the best performance in IBU degradation. This aligns well with the scavenger tests that showed greater involvement of O_2_˙^−^ in the case of 5 nm Au/WO_3_ IO photoanodes (Fig. 8d).

Therefore, two major synergistic mechanisms contribute to the enhancement of photoelectrocatalytic performance in Au/WO_3_ IO photoanodes (Scheme 2): near-field amplification from LSPR that is also assisted by slow photon effects, enhancing e^−^–h^+^ pair generation, and Fermi level alignment-driven electron transfer, enabling effective electron scavenging by Au NPs and facilitating the charge separation. These mechanisms are most effective for the smallest 5 nm Au NPs due to their favourable band alignment and high density of metal–semiconductor interfaces. However, the tendency of smaller NPs to aggregate can red-shift the LSPR band outside the WO_3_ absorption range, limiting near-field effects and PEC performance (Fig. 5).

Maintaining stable PEC performance is crucial for water-splitting and photoelectrocatalytic applications.^32,33,66,67^ For the 20 nm Au/WO_3_ IO photoanodes, prolonged PEC testing at 1.23 *V*_RHE_ showed an initial photocurrent increase to ∼0.125 mA cm^−2^, followed by a stable plateau of ∼0.10 mA cm^−2^ maintained over 4.5 h (Fig. S3, SI). Post-test TEM and EDX analyses confirmed that the Au-decorated WO_3_ IO structure remained intact, demonstrating good structural stability. The durability of the 5 nm Au/WO_3_ IO films was assessed through three consecutive IBU photoelectrocatalytic cycles in 0.1 M NaHCO_3_ at +1.5 V *vs.* Ag/AgCl, with intermediate UV-Vis cleaning (2 h) in deionized water. A modest 13% decrease in the kinetic constant after the third cycle (Fig. S4, SI) indicated appreciable stability, corroborated by post-cycle TEM-EDX analysis showing preserved WO_3_ IO morphology and uniform Au NP coverage.

## Conclusions

This study demonstrated the synergistic interplay between plasmonic, photonic, and charge transfer effects in Au-decorated WO_3_ IO photoanodes for solar-driven water splitting and the photoelectrocatalytic degradation of pharmaceutical contaminants. The well-ordered WO_3_ IO scaffold enabled slow photon effects by aligning its PBG edges with the intrinsic absorption of WO_3_ and the LSPR of Au NPs, thereby amplifying light–matter interactions. The incorporation of plasmonic Au nanoparticles (NPs) significantly enhanced the photoactivity of WO_3_, though with distinct size-dependent behaviour depending on the target application. For PEC water splitting, the optimum performance was achieved with 20 nm Au NPs, where LSPR-induced near-field enhancement led to increased light absorption and charge carrier generation within the WO_3_ photonic scaffold. In contrast, the photoelectrocatalytic degradation of IBU was most efficient with 5 nm Au NPs, despite their lower LSPR contribution. This was attributed to a size-dependent Fermi level shift and reduced Schottky barrier at the Au–WO_3_ interface, which facilitated electron transfer from WO_3_ to Au NPs, promoting charge separation. The combination of plasmonic near-field enhancement, slow photon-induced light trapping, and optimized charge separation led to significantly improved performance in both PEC and photoelectrocatalytic applications. These results underline the importance of rationally engineering light absorption and interfacial charge dynamics in plasmonic–photonic hybrid photoelectrodes, offering a promising strategy for integrated solar energy conversion and environmental remediation technologies.

## Author contributions

Maria-Athina Apostolaki performed experimental investigations, formal analysis, methodology and writing of the original draft. Marios-Konstantinos Christoforou, Elias Sakellis, Polychronis Tsipas, Vassilis Psycharis and Spiros Gardelis contributed to the experimental investigations, formal analyses and results validation. Vlassis Likodimos contributed to the conceptualization, methodology, supervision, resources, writing – review & editing.

## Conflicts of interest

There are no conflicts to declare.

## Supplementary Material

RA-015-D5RA04834F-s001

## Data Availability

The original contributions presented in this study are included in the article and the supplementary information (SI). Further inquiries can be directed to the corresponding author. Supplementary information: linear sweep voltammetry and chronoamperometry for planar films. Tauc plots for WO_3_ IO. Long-term PEC performance and cycling IBU degradation tests. See DOI: https://doi.org/10.1039/d5ra04834f.
