# Supplementary material for: Tailoring plasmonic size in Au/WO3 photonic crystals for photoelectrochemical water splitting and pharmaceutical degradation
Source: RSC Adv. 2025 Nov 4;15(50):42687–97. doi: 10.1039/d5ra04834f (PMC12584951; doi:10.1039/d5ra04834f)
Supplement: RA-015-D5RA04834F-s001 [file RA-015-D5RA04834F-s001.pdf]

Supplementary Information (SI) for RSC Advances.
This journal is © The Royal Society of Chemistry 2025

Electronic Supplementary Information (ESI)

Tailoring Plasmonic Size in Au/WO_3 Photonic Crystals for Photoelectrochemical Water Splitting and Pharmaceutical Degradation

Maria-Athina Apostolaki,[a] Marios-Konstantinos Christoforou,[a] Elias Sakellis,[a,b] Polychronis Tsipas,[b] Vassilis Psycharis,[b] Spiros Gardelis [a] and Vlassis Likodimos *[a]

[a]*Section of Condensed Matter Physics, Department of Physics, National and Kapodistrian University of Athens, University Campus, 15784 Athens, Greece*

[b]*Institute of Nanoscience and Nanotechnology, National Center for Scientific Research "Demokritos", Agia Paraskevi, 15341 Athens, Greece*

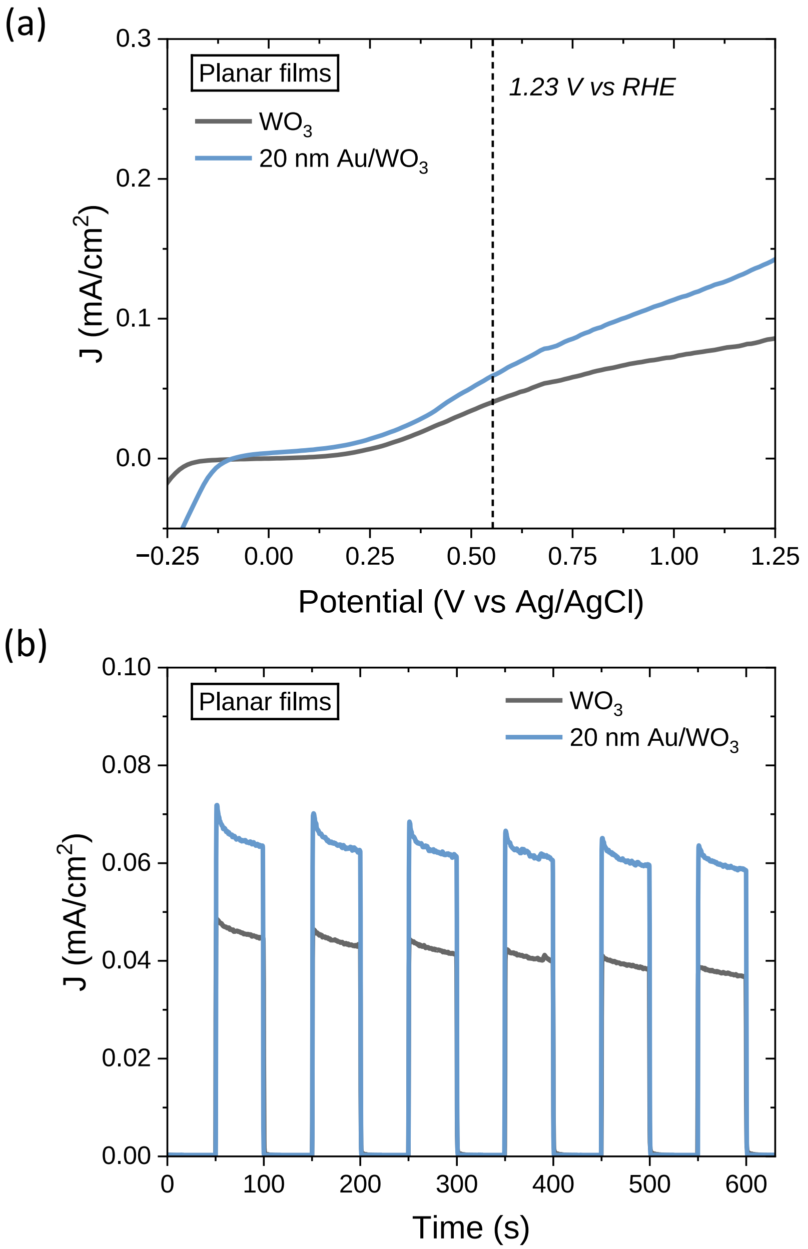

Fig. S1 (a) Current density-potential curves and (b) chronoamperometry under chopped UV–Vis back-side illumination at 1.23 V vs RHE for planar WO_3 and 20 nm Au/WO_3 films. All measurements are carried out in 0.1 M $NaHCO_3$ aqueous electrolyte.

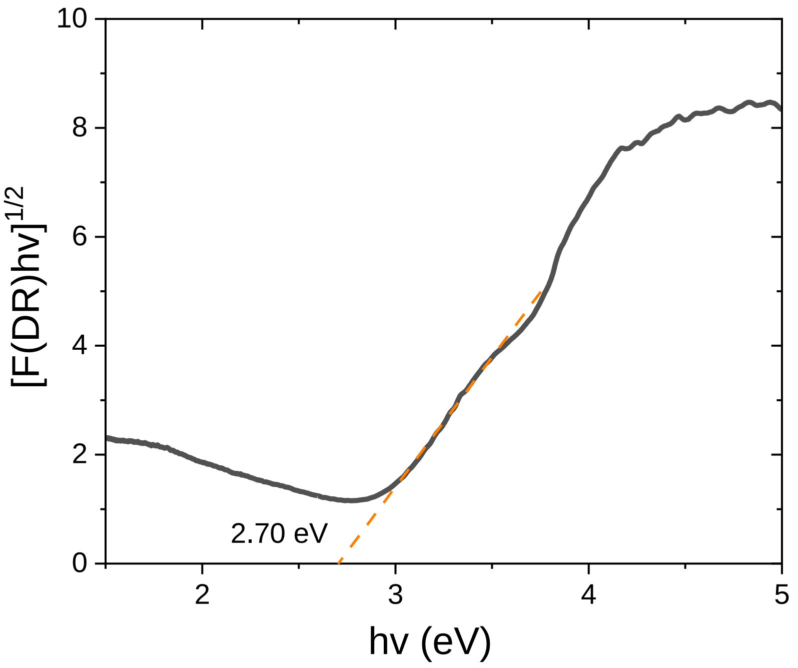

Fig. S2 Indirect band gap Tauc plot for the pristine WO_3 IO film.

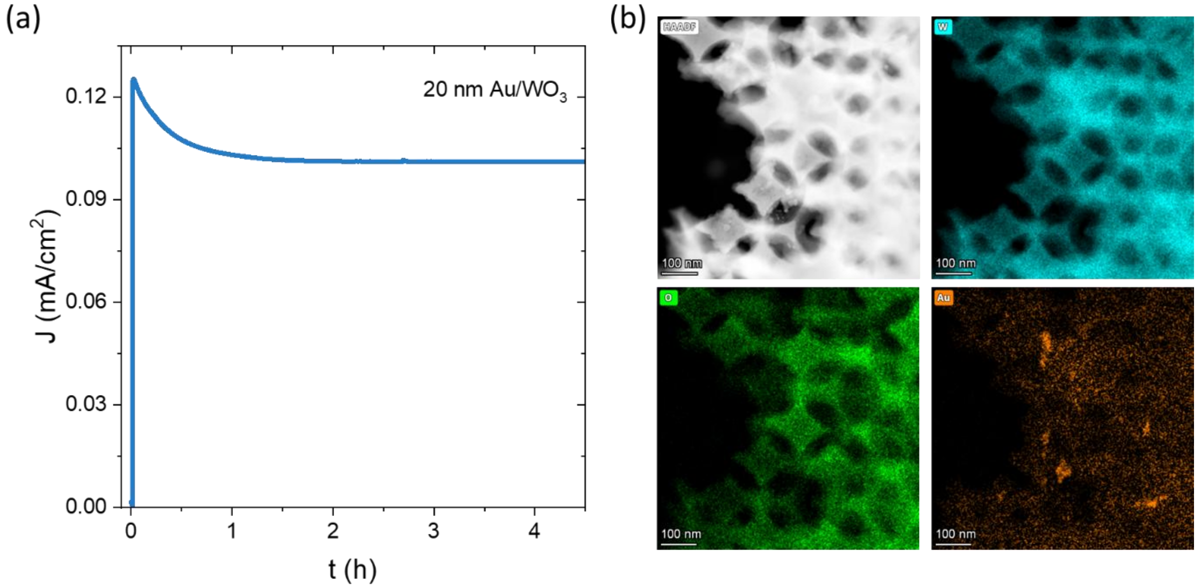

Fig. S3 (a) Chronoamperometric J vs t curves under continuous UV–Vis back-side illumination for 4.5 h at 1.23 V vs RHE for the 20 nm Au/WO_3 IO photoanode. (b) TEM image and EDX elemental (W, O, and Au) maps for the 20 nm Au/WO_3 IO film after 4.5 h.

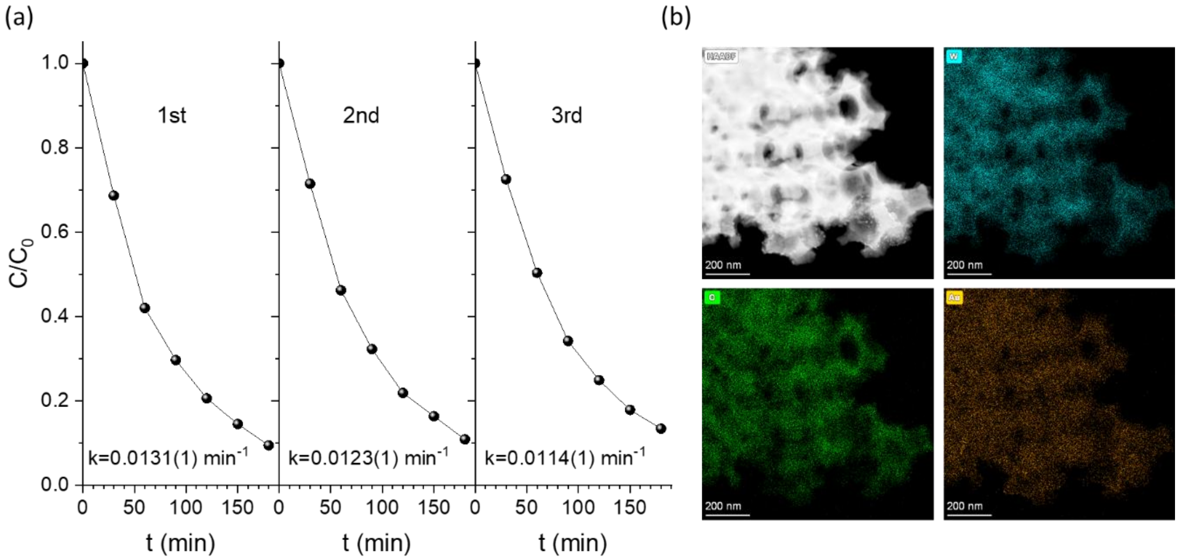

Fig. S4 (a) IBU degradation kinetics for three successive tests using the same 5 nm Au/WO_3 IO photoelectrode in 0.1 M $NaHCO_3$ supporting electrolyte at +1.5 V vs Ag/AgCl under UV–Vis irradiation. (b) TEM image and EDX elemental (W, O, and Au) maps for the 5 nm Au/WO_3 IO film after the 3rd photoelectrocatalytic test.
